# Comprehensive Output Estimation of Double Scattering Proton System With Analytical and Machine Learning Models

**DOI:** 10.3389/fonc.2021.756503

**Published:** 2022-01-31

**Authors:** Jiahua Zhu, Taoran Cui, Yin Zhang, Yang Zhang, Chi Ma, Bo Liu, Ke Nie, Ning J. Yue, Xiao Wang

**Affiliations:** ^1^ Department of Radiation Oncology, Rutgers-Cancer Institute of New Jersey, Rutgers-Robert Wood Johnson Medical School, New Brunswick, NJ, United States; ^2^ Department of Radiation Oncology, Reading Hospital, Tower Health, West Reading, PA, United States; ^3^ Department of Radiation Oncology, City of Hope National Medical Center, Duarte, CA, United States

**Keywords:** output model, analytical model, machine learning, Gaussian process regression, double scattering proton system

## Abstract

**Objectives:**

The beam output of a double scattering proton system varies for each combination of beam option, range, and modulation and therefore is difficult to be accurately modeled by the treatment planning system (TPS). This study aims to design an empirical method using the analytical and machine learning (ML) models to estimate proton output in a double scattering proton system.

**Materials and Methods:**

Three analytical models using polynomial, linear, and logarithm–polynomial equations were generated on a training dataset consisting of 1,544 clinical measurements to estimate proton output for each option. Meanwhile, three ML models using Gaussian process regression (GPR) with exponential kernel, squared exponential kernel, and rational quadratic kernel were also created for all options combined. The accuracy of each model was validated against 241 additional clinical measurements as the testing dataset. Two most robust models were selected, and the minimum number of samples needed for either model to achieve sufficient accuracy ( ± 3%) was determined by evaluating the mean average percentage error (MAPE) with increasing sample number. The differences between the estimated outputs using the two models were also compared for 1,000 proton beams with a randomly generated range, and modulation for each option.

**Results:**

The polynomial model and the ML GPR model with exponential kernel yielded the most accurate estimations with less than 3% deviation from the measured outputs. At least 20 samples of each option were needed to build the polynomial model with less than 1% MAPE, whereas at least a total of 400 samples were needed for all beam options to build the ML GPR model with exponential kernel to achieve comparable accuracy. The two independent models agreed with less than 2% deviation using the testing dataset.

**Conclusion:**

The polynomial model and the ML GPR model with exponential kernel were built for proton output estimation with less than 3% deviations from the measurements. They can be used as an independent output prediction tool for a double scattering proton beam and a secondary output check tool for a cross check between themselves.

## Introduction

Proton therapy is rapidly becoming one of the primary cancer treatment modalities in the recent decade. The utilization of the Bragg peak plays a pivotal role in delivering the prescription dose to the target, while sparing the normal tissues by stopping the proton beam at the distal end of the target (1–4). In order to cover the entire target with a desired dose, the pristine Bragg peak has to be modulated to the spread-out Bragg peak (SOBP) in terms of target size and depth (5–7). Due to the complexity of proton beamline to form various SOBPs in a double scattering proton machine, it is hard to model the output accurately. Therefore, most proton centers with a double scattering beam system have to measure the output of patient-specific proton beams in a water phantom to determine the required machine output, mostly in terms of monitor unit (MU).

In order to obtain the output of a proton beam conveniently and verify the output measurement, Kooy et al. proposed a semi-empirical analytical method to estimate the output as a function of r = (R - M)/M, where R and M denote the beam range and modulation, respectively (8, 9). This formula implements a basic model as a function of r and also corrects for the effective source position based on the inverse square law. However, this model was sensitive to the definition of range and modulation (10). A variation of 18% in output was observed at beam data with small modulation (10, 11). Therefore, Lin et al. proposed a parameterized linear quadratic model which defined r with a limited length of modulation (11). With this correction, the relative errors of predicted outputs compared to measured values were less than 3%. Besides, the basic model of output in Kooy’s method was also fitted by the fourth-order Taylor polynomial multiplied by a range-related factor, which was close to unity (12). In terms of a comparison between Kooy’s original method and the Taylor series approach, the predicted values from the Taylor series approach were closer to the measurements. Sahoo et al. comprehensively analyzed the determination of output from the proton machine beamline, where the relative output factor, SOBP factor, and range shifter factor were the primary factors to determine the output (13). The result also showed a good agreement to the measurement within 2% for 99% of those fields. However, this method required a large amount of measurements to verify the conversion from the SOBP factor and range shifter factor to the output, which was time-consuming and complicated. Machine learning (ML) models have also been used in output prediction (14). Sun et al. compared the accuracy of output from machine learning and Kooy’s method (10). Up to 7.7% of relative error from Kooy’s method was reduced to 3.17% by machine learning.

We propose three analytical models and three machine learning algorithms for output estimation. The analytical models include a polynomial fitting model, a linear fitting model, and a logarithm–polynomial fitting model, all with different equations for different options. The machine learning algorithms utilize the Gaussian process regression (GPR) model with different kernels to test the accuracy of output estimations, with one single model for all options. The definition of R and M is consistent with the machine vendor’s definition, and the data are from our clinical beam measurement. The comparison between predicted and measured outputs was performed. In addition, the minimum number of beam data measurements needed for building a robust model is discussed, which can provide some insights for clinical implementation.

## Materials and Methods

### Introduction of the Proton Machine

Mevion (Mevion Medical Systems, Inc., Littleton, MA) S250 utilizes a double scatter system to broaden the pencil beam and creates a uniform dose distribution with a beam shaping system. The beam shaping system includes primary and secondary scatters, one absorber, and one range modulator, which spread out the Bragg peak. There are two types of nozzles on the inner gantry, a large applicator (maximum 25 cm in diameter) and a small applicator (maximum 14 cm in diameter), respectively. A brass aperture mounted on the applicator shapes the proton beam to cover the target. A compensator mounted at the end of the applicator modulates the distal end of the proton beam. There are 24 options with different beam ranges, beam modulations, and field sizes, as listed in **Table 1**. The first 12 options are large options to be used with the large applicator. The other 12 options are deep/small options to be used with the small applicator.

**Table 1:** The statistics of all options.

	Max range (cm)	Min range (cm)	Max modulation (cm)	Training field	Testing field
Option 1	25.0	22.6	20.0	55	8
Option 2	22.5	20.9	20.0	40	8
Option 3	20.8	18.8	20.0	76	20
Option 4	18.7	16.8	18.7	99	22
Option 5	16.7	14.9	16.7	68	18
Option 6	14.8	13.2	14.8	81	13
Option 7	13.1	11.5	13.1	90	20
Option 8	11.4	10.0	11.4	98	22
Option 9	9.9	8.6	9.9	90	19
Option 10	8.5	7.3	8.5	86	9
Option 11	7.2	6.1	7.2	45	3
Option 12	6.0	5.0	6.0	21	2
Option 13	32.0	29.6	10.0	3	0
Option 14	29.5	27.1	10.0	12	0
Option 15	27.0	24.6	10.0	37	8
Option 16	24.5	22.1	10.0	49	2
Option 17	22.0	20.1	10.0	7	1
Option 18	20.0	17.8	20.0	19	5
Option 19	17.7	15.4	17.7	52	6
Option 20	15.3	13.3	15.3	105	14
Option 21	13.2	11.2	13.2	173	11
Option 22	11.1	9.1	11.1	123	12
Option 23	9.0	7.0	9.0	65	9
Option 24	6.9	5.0	6.9	50	7

### Output Measurement

Due to the complexity of the proton beamline in a double scattering system, the Varian Eclipse treatment planning system (TPS) (Varian Medical Systems, Palo Alto, CA) does not provide MU directly for a proton beam. Instead, the output has to be determined manually for each clinical proton beam.

To determine the MU for a clinical proton beam, a verification plan was generated by copying the original clinical proton beam to a water phantom with the same proton energy fluence. Regardless of the setup in the original clinical plan, a consistent setup with SSD = 190 cm was used in the verification plan. The compensator in the original clinical plan was removed in the verification plan to reduce measurement uncertainty. A reference point was added to determine the dose at the mid-SOBP of the beam, and the output measurements were conducted in the water phantom at the mid-SOBP of the same proton beam with a Farmer chamber (IBA Dosimetry America Inc., Memphis, TN) at SSD = 190 cm. Attention was paid to the in-plane location of the reference point to ensure lateral charge particle equilibrium for accurate dose prediction. Sun et al. and Sahoo et al. demonstrated that the field size effect is negligible with a field opening of at least 5-cm diameter (10, 13). If the verification point was blocked, it was shifted. The same setup was then applied in a water phantom for absolute dose measurement. The absolute point dose at the verification point of 100 MU was measured following the IAEA TRS398 protocol (15). The output was essentially d/MU, where d was the absolute point dose measured, and MU was 100. Given the outputs were the same for both clinical beam and verification beam, the MU of the patient-specific beam would be calculated by taking the ratio of the verification point dose from TPS to the measured output.

### Analytical Model-Based Output Estimation

**Figure 1:**
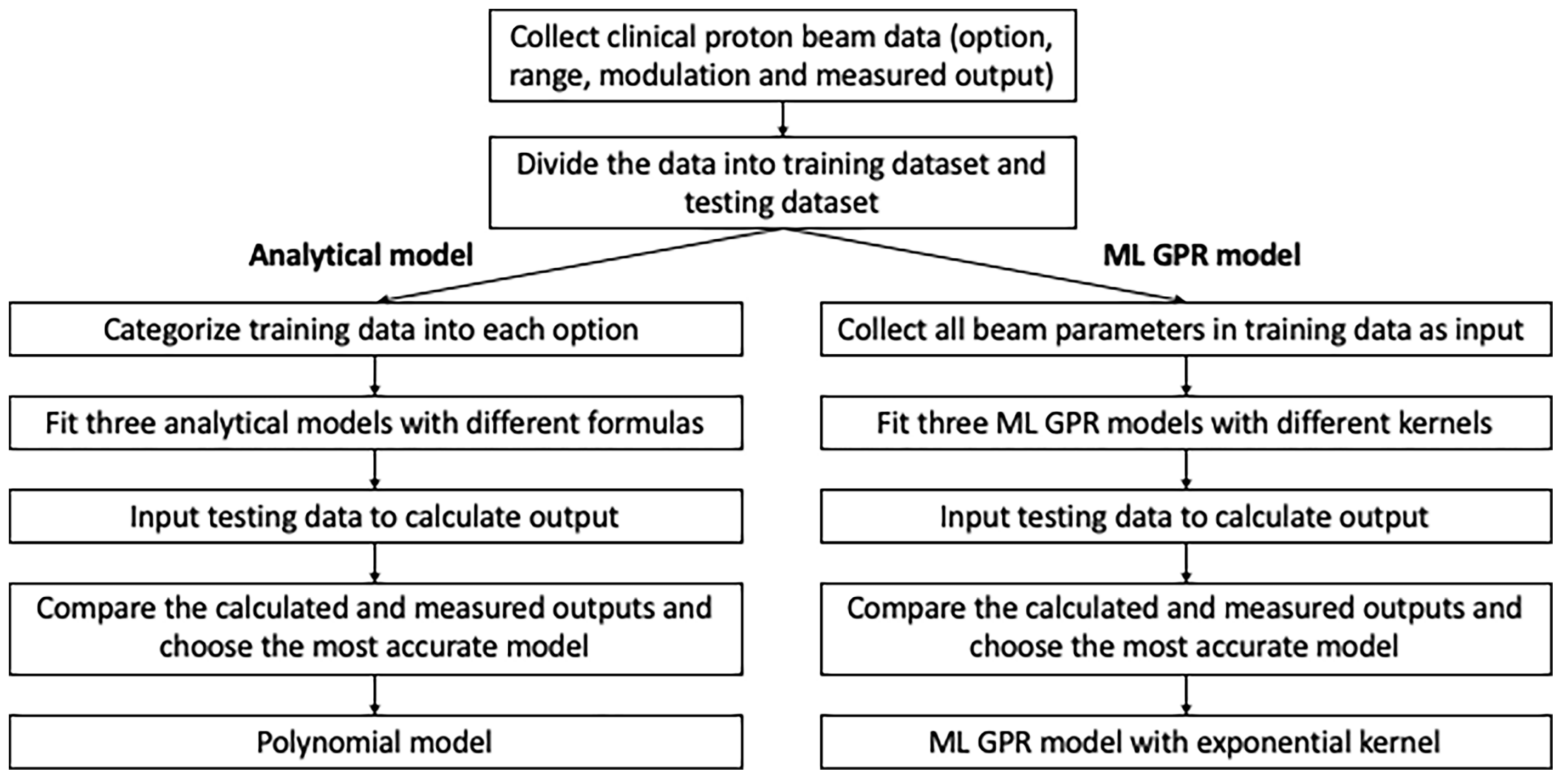
Workflow of model fitting and testing for analytical/ML models.

In order to validate and verify and eventually replace the manual measurement, output models were built based on previous measurements. Analytical models using an empirical formula to convert from range/modulation to output were built for each option, based on 1,785 proton clinical field measurements. 1,544 clinical proton fields from 2015 to 2019 were categorized as training dataset and the rest (241 fields) as the testing dataset. Three analytical models were employed to estimate the output and compared to the measurement as reference. The specific workflow of data modeling and accuracy verification is shown in **Figure 1**.

#### Polynomial Fitting Model

The polynomial fitting model is an adaptation of Kooy’s empirical formula. A simple demonstration is shown for better understanding. In Kooy’s formula (9), output is a function of r = (R - M)/M. According to the vendor definition, R is defined as the depth at distal 90% of the normalized percent depth dose and M is defined as the length between proximal 95% and the distal 90% of the normalized percent depth dose. The basic model of Kooy’s formula is expressed in Ferguson et al. (12) as


(1) $$d/MU(r(R,M))=\frac{CF\times \psi_{c}\times D_{c}}{100/(1+a_{0}r^{a1})}\times [s_{0}+s_{1}(R-R_{L})]\times {\left(\frac{ESAD(r)-\Delta z_{p}}{ESAD(r)-\Delta z_{p}-\Delta z}\right)}^{2}$$

The first term of Eq. 1 is the basic output prediction; the second term corrects the variation of output related to the virtual source position; and the third term is inverse square related.

A polynomial equation of each option was fitted to replace the basic model in Eq. 2 (12):


(2) $$d/MU(r(R,M))=(p_{0}+p_{1}r+p_{2}r^{2}+p_{3}r^{3}+p_{4}r^{4})\times [s_{2}+s_{3}(R-R_{L})]\times {\left(\frac{ESAD(r)-\Delta z_{p}}{ESAD(r)-\Delta z_{p}-\Delta z}\right)}^{2}$$

where s_2_ and s_3_ are the option-specific fitting parameters.

In terms of the fitting data, Ferguson et al. listed the values of s_0_ and s_2_ in different options and those are very close to unity (12). s_1_ and s_3_ were found to be much less than s_0_ and s_2_; therefore, the variation of second terms from unity could be negligible. The third term is only to correct the measurement position if the effective source is not located at the middle SOBP.

To simplify the calculation of output, we replaced the SAD setup with the SSD setup in our method, in which Δz is always zero and the third term equals unity.

Therefore, the equation of output estimation can be approximated by a quadratic Taylor polynomial in Eq. 3.


(3) dMU=a×r2+b×r+c,

where *r* = (*R – M*)/*M*, d/MU denotes the output and a, b, and c are the fitting parameters.

#### Linear Fitting Model

**Figure 2:**
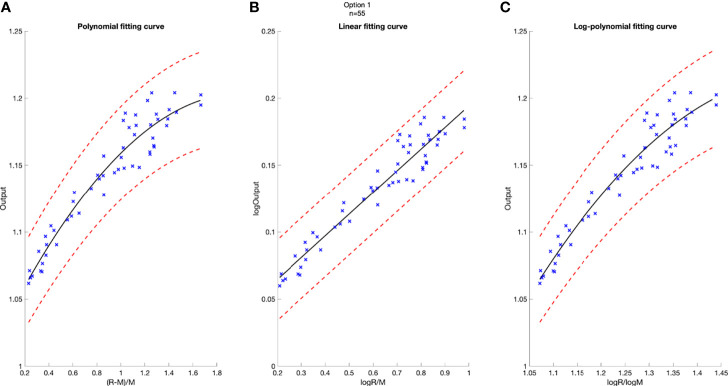
Model-based fitting curves for Option 5, including the polynomial fitting curve **(A)**, the linear fitting curve **(B)**, and the log-polynomial fitting curve **(C)**. 3% confidence level in red dashed line. Number of data points n = 68.

The linear fitting model estimates output as the function of the logarithm of R/M (Eq. 4). The rationale of choosing this model was to space out data points clustered in the low R/M region, as observed from the polynomial model. From the polynomial fitting graph, it was observed that the output variations in the low R/M region (full modulation) were larger, with a lot more data points than the high R/M region (**Figure 2**). This finding is consistent with what Sun et al. and Kim et al. reported (10, 16). This model can be expressed as


(4) log(dMU)=k×log(RM)+b

where d/MU, R, and M are the variables, and k and b are the fitting parameters.

#### Logarithm–Polynomial Fitting Model

The logarithm–polynomial fitting algorithm is an independent model from the previous two models, since the variables in previous models are both related to R/M. To build a model with a different variable, while still keeping the model accurate, different approaches were made and the most accurate one was selected. In this model, the output is the function of logarithm(R)/logarithm(M) in Eq. 5.


(5) dMU=a'×r'2+b×r'+c',

where *r*′ = log(*R*)/log(*M*), and a′, b′, and c′ are the fitting parameters.

### Machine Learning-Based Output Estimation

Different from analytical methods, machine learning (ML) methods do not need to model option by option. Instead, they use option number, beam range, and modulation to predict the output. To test the efficacy and accuracy of ML modeling, three ML GPR models with different kernels, including exponential kernel, squared exponential kernel, and rational quadratic kernel, were used for the output calculation (17). The model is shown in Eq. 6.


(6) y=h(x)β+f(x)

where h(x) is a set of basic functions that transform the original feature vector x into a new feature vector h(x), and f(x) models the uncertainties from the system.

GPR is a non-parametric Bayesian approach toward regression problems that can be utilized in exploration and exploitation scenarios (17, 18). It predicts the output data by incorporating prior knowledge and fit a function to the data. The Gaussian process is a set of random variables, such that any finite number of them has a joint Gaussian distribution. The mean function of f(x) is 0, and the covariance function is k(x, x′), that is, f(x)~GP(0, k(x, x′)).

The probability distribution of y is


(7) $$P(y_{i}|f(x_{i}),x_{i})∼N(y_{i}|h{\left(x_{i}\right)}^{T}\beta +f(x_{i}),\sigma^{2})$$

which can be written in matrix form as


(8) $$P(y|f,X)∼N(y_{i}|H\beta +f,\sigma^{2}I)$$

where


(9) $$X=\left(\begin{array}{c} x_{1}^{T}\\ \vdots \\ x_{n}^{T} \end{array}\right),y=\left(\begin{array}{c} y_{1}\\ \vdots \\ y_{n} \end{array}\right),H=\left(\begin{array}{c} h(x_{1}^{T})\\ \vdots \\ h(x_{n}^{T}) \end{array}\right),f=\left(\begin{array}{c} f(x_{1})\\ \vdots \\ f(x_{n}) \end{array}\right)$$

Then,


(10) P(f|X)∼N(f|0,K(X,X))

K is the covariance matrix


(11) $$K=\left(\begin{array}{ccc} k(x_{1},x_{1}) & \cdots & k(x_{1},x_{n})\\ \vdots & \ddots & \vdots \\ k(x_{n},x_{1}) & \cdots & k(x_{n},x_{n}) \end{array}\right),$$

where k is the kennel function.

The kernels play very significant roles in the regression modeling and can map the features from the original values to the featuring spaces by involving the latent variables. In this model, three kernel functions were used, including exponential kernel, squared exponential kernel, and rational quadratic kernel.

After the training process, 5-fold cross-validation was performed to prevent overfitting. Then, the obtained models were evaluated using the parameters from the testing sets.

### Robustness of Models Related to Sampling Numbers

The robustness of output models could be impacted by the number of data fed into the model. The evaluation of minimum number of data necessary for a robust model was conducted by comparing different model outputs with increasing number of inputs. Models were built with different sampling numbers, randomly selected from the original training dataset. The sampling numbers ranged from 10 to the number of training datasets. Each time a new model was generated, and the mean average percentage error (MAPE) was calculated to evaluate the differences between predicted outputs and the corresponding measurements. The comparisons were performed per option for polynomial models.

### Difference Between Analytical and ML Models

Analytical fitting models and ML models play independently in output estimation, as can be seen in **Figure 1**. Although both polynomial model and ML GPR model with exponential kernel may be robust and accurate enough to be within clinical tolerance, the predicted output from the two models can be different. Also, a cross check of output is essential to verify the accuracy and effectiveness of the two methods. To assess the difference between the two models, 1,000 random points within the range and modulation of each option were generated to estimate output by using these two models, and the MAPE of predicted outputs and the corresponding measurements for the two models were calculated for each point. The estimated output with different combinations of range and modulation were compared between these two models and also to the measured output.

## Results

The total clinical fields including training data and testing data were categorized into 24 options (**Table 1**). In this table, Options 4, 6, 7, 8, 9, 10, 20, 21, and 22 were mostly used in clinic with sample numbers larger than 80.

### Accuracy Analysis of Output Estimation

A deviation of 3% was used as tolerance in clinical output estimation. The analytical fitting curve of Option 5 is presented as an example in **Figure 2** to show the absolute error of modeling output relative to the measured value. The output is plotted as a function of R and M, with polynomial fit in **Figure 2A**, linear fit in **Figure 2B**, and logarithm–polynomial fit in **Figure 2C**. The red dashed line represents ±3% from the predicted output. The blue scattered marks representing the real measurements are all within ±3% of the predicted value in Option 5, indicating accurate prediction for three models. The coefficient of determination of each fitting curve is provided on **Figure 2**.

**Figure 3:**
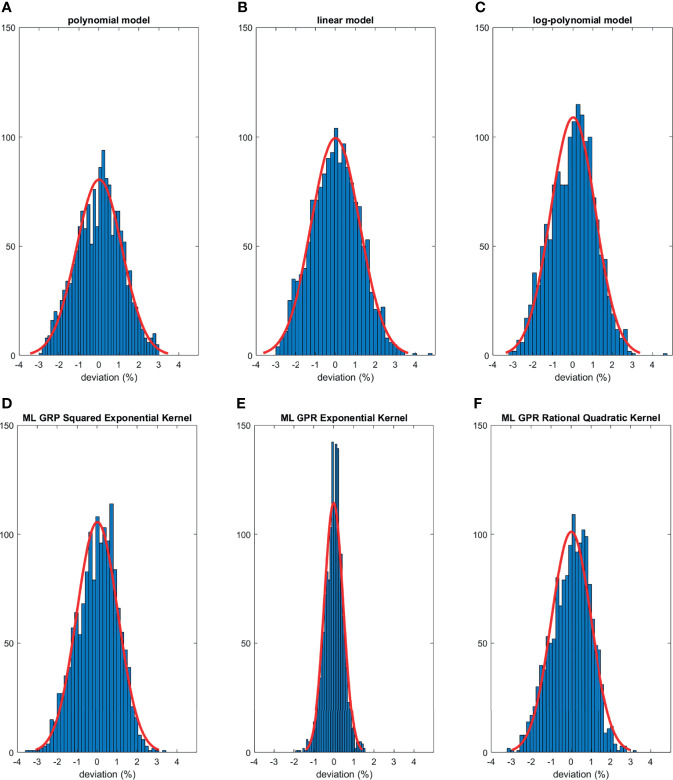
Histograms of percent difference between analytical/ML GPR models and measurements using training data.

**Figure 4:**
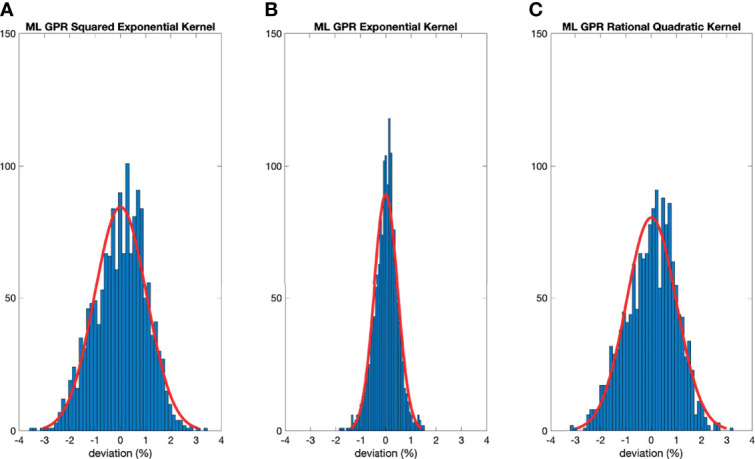
Histograms of percent difference between the ML GPR models and measurements using 5-fold cross-validation.

**Figure 5:**
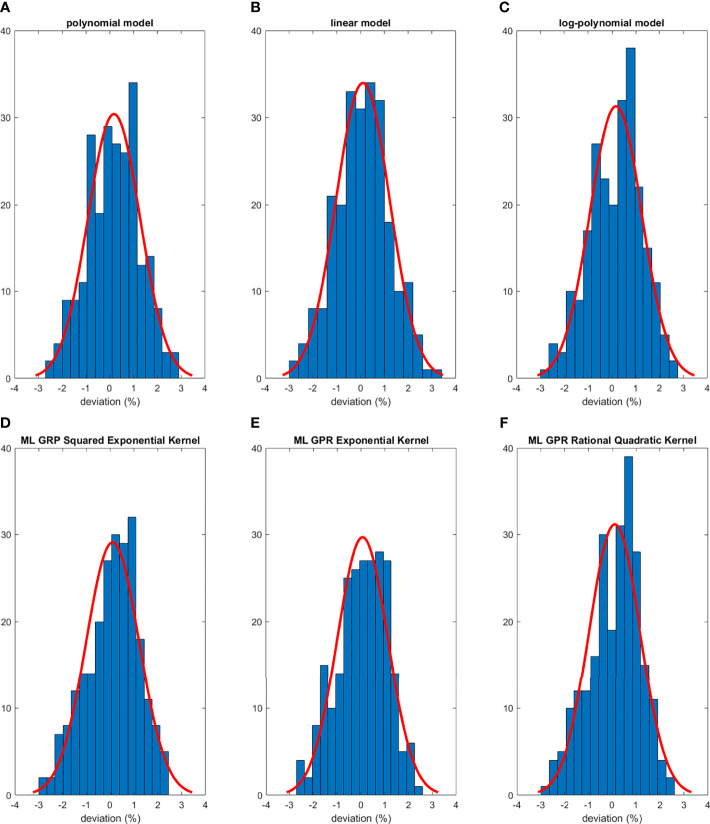
Histograms of percent difference between analytical/ML models and measurements using testing data.

The histograms of the relative deviation of all 24 options are categorized in **Figure 3**. Compared to the other two analytical models, the polynomial fitting model provided a better agreement with measurement data, with all deviations within ±3%. In ML GPR models, the exponential kernel showed a more accurate output estimation than the other two with less than ±2% deviation from the measurement. The 5-fold cross-validation results are shown in **Figure 4**, which showed a similar performance as the training model. In addition, the testing data were imported into analytical and ML GPR models to verify the effectiveness and accuracy of output estimation (**Figure 5**). It was observed that the polynomial fitting model still provided a good output estimation within 3% deviation, and all the ML models also exhibited deviation within ±3%. To summarize, the polynomial model and ML GPR model with exponential kernel showed the best performance among all 6 models, with less than ±3% deviation from all measurement data.

### Minimum Number of Fields Needed for Polynomial Model and ML GPR Model With Exponential Kernel Model

**Figure 6:**
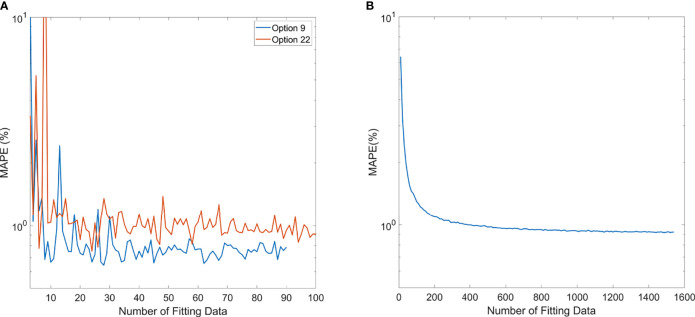
Mean average percentage error (MAPE) of polynomial model **(A)** and ML exponential kernel model **(B)** with the increase in fitting data.

The trend of MAPE of models compared to measurements is shown in **Figure 6**. For the polynomial model, since it is specific to each option, Option 9 and Option 22 were chosen as the representatives of the polynomial model because of higher sample numbers available, as shown in **Figure 6A**. The trend of MAPE for the ML GPR model with the exponential kernel is shown in **Figure 6B**, with data from all options. As observed in this figure, the relative error in Options 9 and 22 both converged to be around 1% or less once 20 data points were used for building the polynomial model. For the ML GPR model with the exponential kernel, the convergence of MAPE was reached at around 400 data points, regardless of the option.

### Evaluation of Difference Between Analytical Models and ML Models

**Figure 7:**
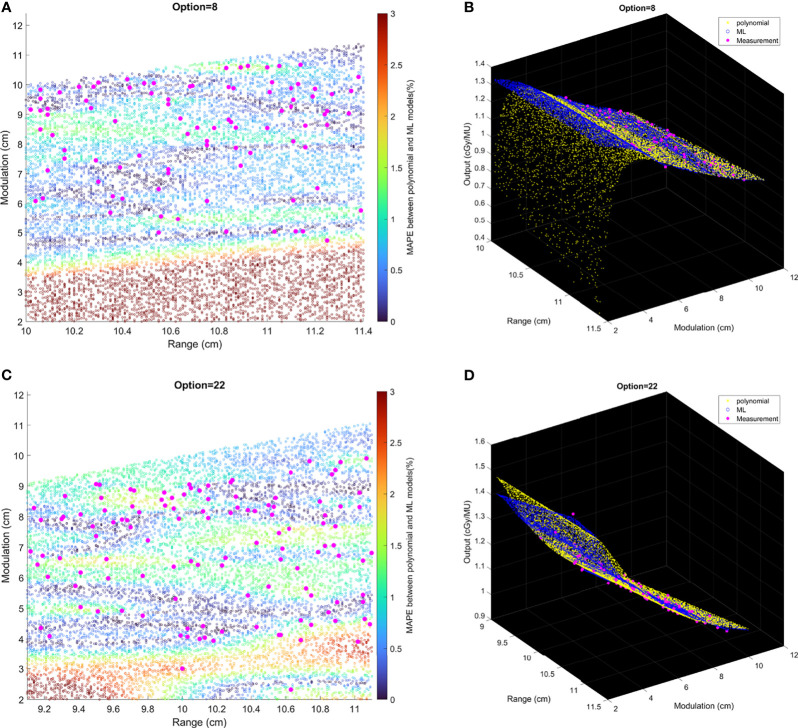
The relative difference of output estimation with R and M. **(A, C)** show the differences of two models. The solid pink points are the measurement data, and the circles are MAPE between two models from the random data. **(B, D)** are the differences in 3D graphs that illustrate the trend of difference between two models.

Comparisons of output estimation between the polynomial fitting model and the ML GPR model with the exponential kernel for Options 8 and 22 are shown in **Figure 7**. MAPE for 1,000 randomly generated points between the two models are shown with corresponding R and M. Measurement data are marked as pink scattered points overlaid on the figure. It is shown in **Figure 7** that the two models agreed well in the regions where there were measurement data, with MAPE less than 2%. Considerable differences in the outputs were observed beyond the measurement region. More intuitive figures are shown in **Figure 7B** where the general trend of outputs splits in between polynomial and ML models with the decrease of modulation, and this split in 3D graphs illustrates the trend of difference between the two models.

## Discussion

Patient-specific output estimation in a double scattering proton machine has to rely on manual measurement, which can be a labor-intensive and error-prone process, so it is valuable to build models to second-check manual measurement, and the ultimate goal of the study is to build an automatic output estimation and MU determination process.

The output estimation derived from three analytical fitting models and three ML GPR models with different kernels was demonstrated and compared to the measurements. The polynomial fitting model and ML GPR model with the exponential kernel with the best performance were chosen. In terms of the distribution in the histogram, the polynomial fitting method provided the most accurate output estimation in analytical methods and the ML GPR model with the exponential kernel could provide more accurate output prediction than the other two ML GPR models. Also, the relative errors between the estimated and measured output for the polynomial fitting model and ML GPR model with exponential kernel were always within ±3% in both training data and testing data. Therefore, it is proof that those two models could be adopted as the output estimation models. Also, since they are two independent models, it is suggested that they can be used as second-check tools for clinical measurement, and also a cross-check tool for each other.

Compared with other models reported in literature, our models are more stable and accurate in output estimation. It has been reported by Kooy et al. (8, 9) that there was a large deviation between calculated and measured output in full range and full modulation. Sun et al. (10) also reported an apparent difference (>3%) even using their ML models. Comparatively, the advantage of our polynomial model and the ML GPR model is that the difference between the measured and calculated output is within 3%, which satisfies the clinical requirement and is thereby reliable for clinical use.

The polynomial model is an expansion of Kooy’s empirical formula using Taylor series. This approach is similar to the equation developed by Ferguson et al. (12) but using a lower order of polynomials, thus simplifying the equation. As shown in the results, the performance of our quadratic polynomial model is comparable to their quartic polynomial model, as both models can achieve an accuracy of ±3%. The reason for the polynomial model to outperform the logarithm–polynomial model is probably because the variable of the polynomial model is *r* = (*R*–*M*)/*M*, same as that in Kooy’s empirical model (8), which is a theoretical equation derived from physical properties of proton beam lines, while the variable of the logarithm–polynomial model is *r′* = log(*R*)/log(*M*). The result of better performance of the polynomial model proves that the proton output is truly related to *R*/*M*. The intention of building the linear model was to space out data points clustered in the low *R*/*M* region, as observed from the polynomial model, to avoid overfitting. However, as the results show, the linear curve cannot simulate the trend of training data, thus resulting in a larger deviation. As a result, the other two analytical models cannot predict the output with similar accuracy as the polynomial model.

The reason to choose the GPR model for the output prediction is that the kernel functions can be used. Prior knowledge and specifications about the shape of the model can be added by selecting different kernel functions. Meanwhile, the Gaussian process directly captures the model uncertainty. In this way, the output model can be described as a distribution rather than approximated values. The exponential kernel was based on the assumption that the Euclidean distances between different data points were Laplacian distributed. The squared exponential kernel used another assumption that the Euclidean distances were normally distributed. The rational quadratic kernel can be seen as a scale mixture of squared exponential kernels with different characteristic length-scales (17). In this paper, the distributions of modulations and ranges were close to sparse, so the Laplacian distribution might be a better option, resulting in that the exponential kernel exhibited better performance.

The number of data needed for establishing a robust polynomial model was estimated by the MAPE trend with increasing data points. Options 9 and 22 were shown as an example that the MAPE converged once the number of training data increased to 20. This gives a simple guidance on the number of data necessary to build an accurate polynomial model for an option. Among all options, some of them were rarely used, especially the deep options (option 13 with 3 beams, option 14 with 12 beams, option 15 with 37 beams, option 17 with 7 beams, and option 18 with 19 beams). This is because clinically we tend to plan the proton beam to penetrate through a shorter path if possible, leading to lower usage of deep-ranged options. For those options with fewer data points, a polynomial-based output model would not be recommended. Instead, manual measurement would be required, until enough data points are accumulated.

For the ML GPR model with exponential kernel, convergence of MAPE to 1% was observed after the input of 400 fields. This needs to be clarified that when building the ML models, range and modulation as well as the option number were inputs to the model. Sun et al. also estimated the minimum number of fields needed for the ML cubist model (10). In their study, the mean absolute error converged to 0.7% after 1,200 data points. Their learning curves also showed a mean absolute error around 1% at 400 samples. Since the ML model does not discriminate different options, and some options may have fewer data samples than others, validation of accuracy of the model in all options is needed before clinic implementation.

The polynomial fitting model and ML method could be used as independent secondary check, and eventually the primary output estimation, replacing measurements. This requires the assessment of the agreement between two models. From **Figure 7**, the MAPE between two models was less than 2% if the data points lay within the region where there existed measurement data. Beyond this region (e.g., M = 2–6 cm in Option 4), the two models showed obvious different trends with increasing differences, which indicated that the user must evaluate the accuracy of output prediction with extra measurements; otherwise, the model cannot be used beyond the region with real measurements. It is suggested that the models should only be trusted to replace measurements with judgment that the beamline (R and M) falls within the region with enough measurement data.

Even though the polynomial fitting model and ML GPR model with the exponential kernel proved their feasibility for output estimation, it is still essential to pay attention to MU determination, as not only is the MU related to output but also its accuracy is related to the verification point dose. The accurate selection of the verification point is pivotal in MU determination. It is recommended to perform a sanity check on the MU of a clinical plan. A simple sanity check is to compare calculated MU to the prescription dose multiplied by the field weighting. The rationale of this sanity check is because the output is always close to 1. Future work includes automatic MU determination with Eclipse Scripting, to help get rid of the uncertainty of manual selection of verification points. Nevertheless, whether the output is measured or modeled, the MU must be verified prior to clinical treatment.

## Conclusions

MU determination including output measurement is one of the most time-consuming and complicated works in patient QA for double-scattering proton machines. Compared with the output measurement, analytical fitting models or ML models are more efficient to provide output estimation. Out of the six models presented in this paper, the polynomial model and ML GPR model with the exponential kernel both show accurate estimation, and the accuracy meets the clinical requirement (within ±3%). The minimum number of data needed to build a robust model is provided, although it is suggested that the validation of accuracy of the model is needed before clinical implementation. These independent output estimations can serve as second-check tools for measurements and have potential to replace the measurement as part of the standard MU determination procedure. The models exhibit robustness within the region where there exist measurement data, and the accuracy beyond the region with real measurements must be evaluated with extra measurements.

## Data Availability Statement

The raw data supporting the conclusions of this article will be made available by the authors, without undue reservation.

## Ethics Statement

Ethical review and approval were not required for the study on human participants in accordance with the local legislation and institutional requirements. Written informed consent for participation was not required for this study in accordance with the national legislation and the institutional requirements.

## Author Contributions

XW, YiZ, and KN led the conception and design of the study. JZ, XW, BL, YiZ, TC, and KN contributed to the acquisition of data. JZ, XW, TC, YaZ, and CM contributed to the analysis, data modelling, and interpretation of data. JZ, XW, TC, and NY drafted and revised the article. All authors contributed to the article and approved the submitted version.

## Conflict of Interest

The authors declare that the research was conducted in the absence of any commercial or financial relationships that could be construed as a potential conflict of interest.

## Publisher’s Note

All claims expressed in this article are solely those of the authors and do not necessarily represent those of their affiliated organizations, or those of the publisher, the editors and the reviewers. Any product that may be evaluated in this article, or claim that may be made by its manufacturer, is not guaranteed or endorsed by the publisher.
